# Mobile Health Intervention in the Maternal Care Pathway: Protocol for the Impact Evaluation of hAPPyMamma

**DOI:** 10.2196/19073

**Published:** 2021-01-19

**Authors:** Manila Bonciani, Sabina De Rosis, Milena Vainieri

**Affiliations:** 1 Management and Healthcare Laboratory Institute of Management and Department EMbeDS Sant’Anna School of Advanced Studies Pisa Italy

**Keywords:** mHealth, maternal care pathway, impact evaluation, quasiexperimental study

## Abstract

**Background:**

Mobile health (mHealth) has great potential to both improve the quality and efficiency of care and increase health literacy and empowerment of patient users. There are several studies related to the introduction of mHealth tools for supporting pregnancy and the postnatal period, with promising but not yet rigorously evaluated impacts. This article presents the protocol for evaluating an mHealth intervention (hAPPyMamma) applied in the maternal and child care pathway of a high-income country (in a pilot area of Tuscany Region, Italy).

**Objective:**

The protocol describes hAPPyMamma and the methods for evaluating its impact, including the points of view of women and practitioners. The research hypothesis is that the use of hAPPyMamma will facilitate a more appropriate use of available services, a better care experience for women, and an improvement in the maternal competencies of the women using the app compared to the control group. The protocol also includes analysis of the organizational impact of the introduction of hAPPyMamma in the maternal pathway.

**Methods:**

A pre-post quasiexperimental design with a control group is used to undertake difference-in-differences analysis for assessing the impact of the mHealth intervention from the mothers’ points of view. The outcome measures are improvement of maternal health literacy and empowerment as well as experience in the maternal care pathway of the control and intervention groups of sampled mothers. The organizational impact is evaluated through a quantitative and qualitative survey addressing professionals and managers of the maternal care pathway involved in the intervention.

**Results:**

Following study recruitment, 177 women were enrolled in the control group and 150 in the intervention group, with a participation rate of 97%-98%. The response rate was higher in the control group than in the intervention group (96% vs 67%), though the intervention group had less respondent loss at the postintervention survey (10% compared to 33% of the control group). Data collection from the women was completed in April 2018, while that from professionals and managers is underway.

**Conclusions:**

The study helps consolidate evidence of the utility of mHealth interventions for maternal and child care in developed countries. This paper presents a protocol for analyzing the potential role of hAPPyMamma as an effective mHealth tool for improving the maternal care pathway at individual and organizational levels and consequently helps to understand whether and how to scale up this intervention, with local, national, and international scopes of application.

**International Registered Report Identifier (IRRID):**

DERR1-10.2196/19073

## Introduction

### Background

Mobile health (mHealth) can play a disruptive role in transforming health promotion and health care service provision. mHealth refers to health-related practice supported by mobile and wireless devices, such as mobile phones, smartphones, and tablets, including mobile apps [1]. The growing spread of mobile devices has pushed the use of apps providing digital services, including for health care.

mHealth has great potential to both improve quality and efficiency of care [2,3] and to increase health literacy and empowerment of patient users [4]. Using mHealth apps, people can manage their health and well-being more actively and consciously [3]. Due to its characteristic of ubiquity and the possibility of personalization, it is expected to be a powerful tool for patient-centered care.

Nevertheless, there is contradictory evidence about the impacts of mHealth interventions on health promotion practice and health outcomes [5-8]. There are several studies focusing, in particular, on the introduction of mHealth tools for supporting pregnancy and the postnatal period, with promising but not yet rigorously evaluated impacts [9]. The evidence for effectiveness in behavioral change is also inconsistent, with both ineffective [10] and effective interventions [11,12] targeted at pregnant women and mothers. Furthermore, there is evidence of demonstrated positive outcomes from mHealth tools for pregnant women and future mothers, but also of the difficulties related to the routine integration of mHealth tools into established prenatal and newborn health services [13]. Nonetheless, it is worth pointing out that most of the research studies on mHealth interventions in the field of maternal, neonatal, and newborn care have been undertaken in low- and middle-income countries [14-29].

This protocol presents an mHealth intervention for maternal and child care in a high-income country, including the methods adopted for evaluating its impact at individual and organizational levels.

### Study and Policy Context Concerning mHealth and the Maternal Care Pathway

The context of this study is the region of Tuscany (Italy), which shows characteristics of eHealth diffusion in line with both the national and wider European contexts [30,31].

Within the framework of the Italian public health care system ensuring universal health care coverage, maternal care is guaranteed for all women free of charge as an essential level of care [32,33]. Although this includes services provided by hospitals and family care centers along the entire maternal journey until the postpartum period, the majority of women prefer to be supported by a private gynecologist during pregnancy [34]; this may limit communication of the publicly available community services offered to pregnant women and new mothers. To standardize the prenatal visits and treatments within its territory, the Tuscany Region provides women with a pregnancy booklet, delivered by a midwife at the family care center. Despite such efforts to strengthen the maternal care pathway, some critical issues remain unresolved in Tuscany [35-38]. A first problem concerns the lack of coordination between services, especially where the maternal care pathway requires integration between local health authorities (LHAs) and teaching hospitals. A second issue is related to the communication channels: Tuscan women clearly expressed the preference to be informed using information and communication technology (ICT) such as text messages or emails [35]. A third issue concerns poorer health care service use by specific categories of women, such as foreign women and those with a low level of education [36]. Due to their weaker health literacy, these groups are often the least likely to exercise choice and to have a direct and appropriate relationship with health care services and consequently, equal access to antenatal and postnatal care [39]. A final issue regards infant vaccination coverage, which in 2016 was <90% for measles [40].

To address these weaknesses and gaps, Tuscany financed and promoted the development and pilot study of the mobile app hAPPyMamma in an LHA. hAPPyMamma was designed as a supportive tool for women and professionals who can provide women with information about the maternal care pathway and its services, also targeted at specific categories (such as low-income women). This may increase the opportunity for contact and interaction as well as women’s self-management and can facilitate disadvantaged groups in accessing and using services [41].

### Design and Implementation of the Mobile App hAPPymamma

**Figure 1:**
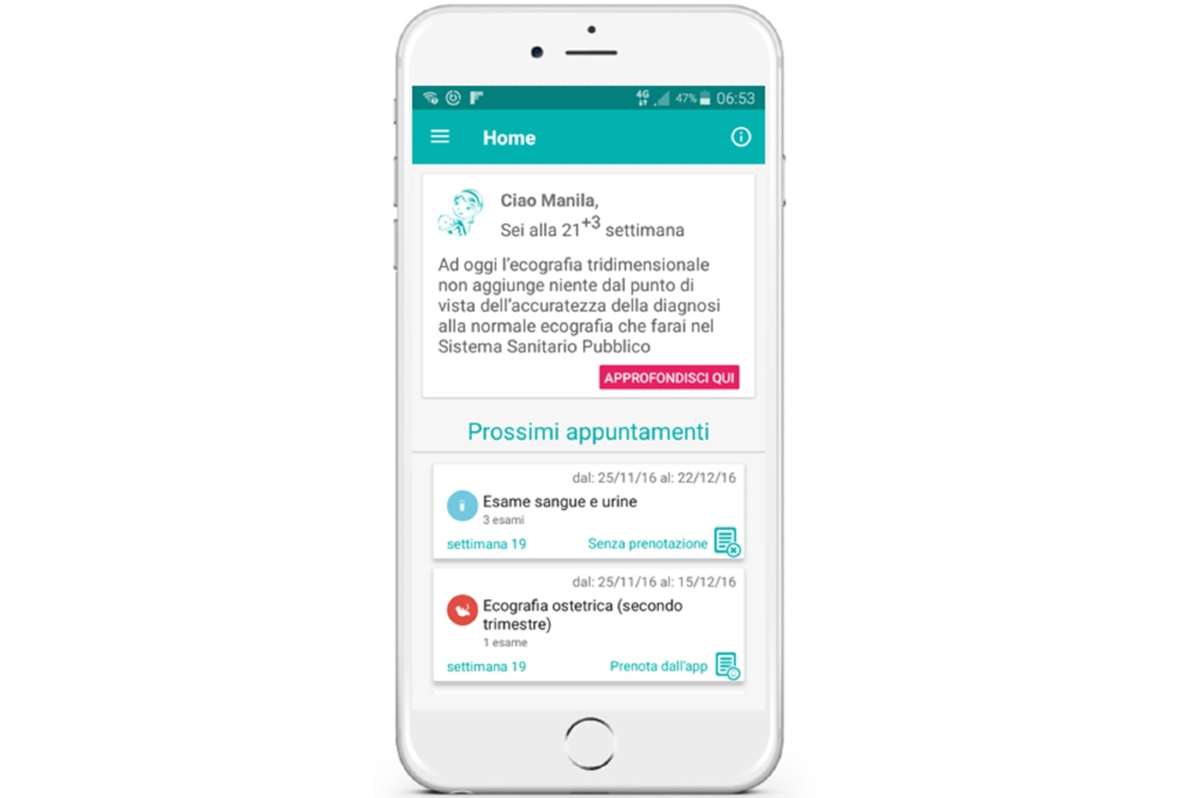
hAPPyMamma home page: identification of gestational age (during pregnancy) or newborn age (in the postpartum period), specific week-by-week messages, and memos for upcoming appointments.

**Figure 2:**
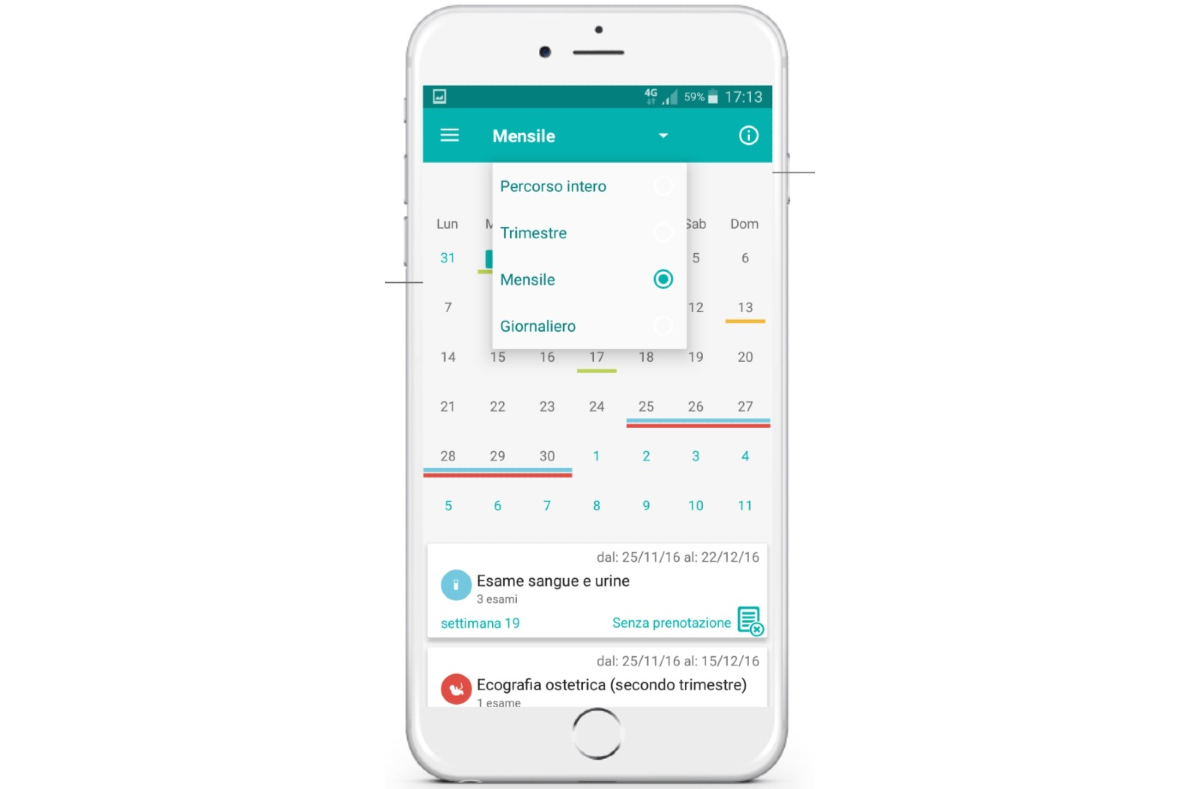
hAPPyMamma agenda: digitization of the pregnancy booklet and infant vaccination calendar in the app planner, with different visualization options and alert mechanisms.

The mobile app offers different functionalities. From the home page, gestational age (during pregnancy) or newborn age (in the postpartum period) are addressed with personalized messages (Figure 1). It includes a digital translation of the pregnancy booklet and infant vaccination calendar into the planner within the app (Figure 2), including alert mechanisms to notify women about visits and diagnostic tests to reserve or, if reserved, to attend.

**Figure 3:**
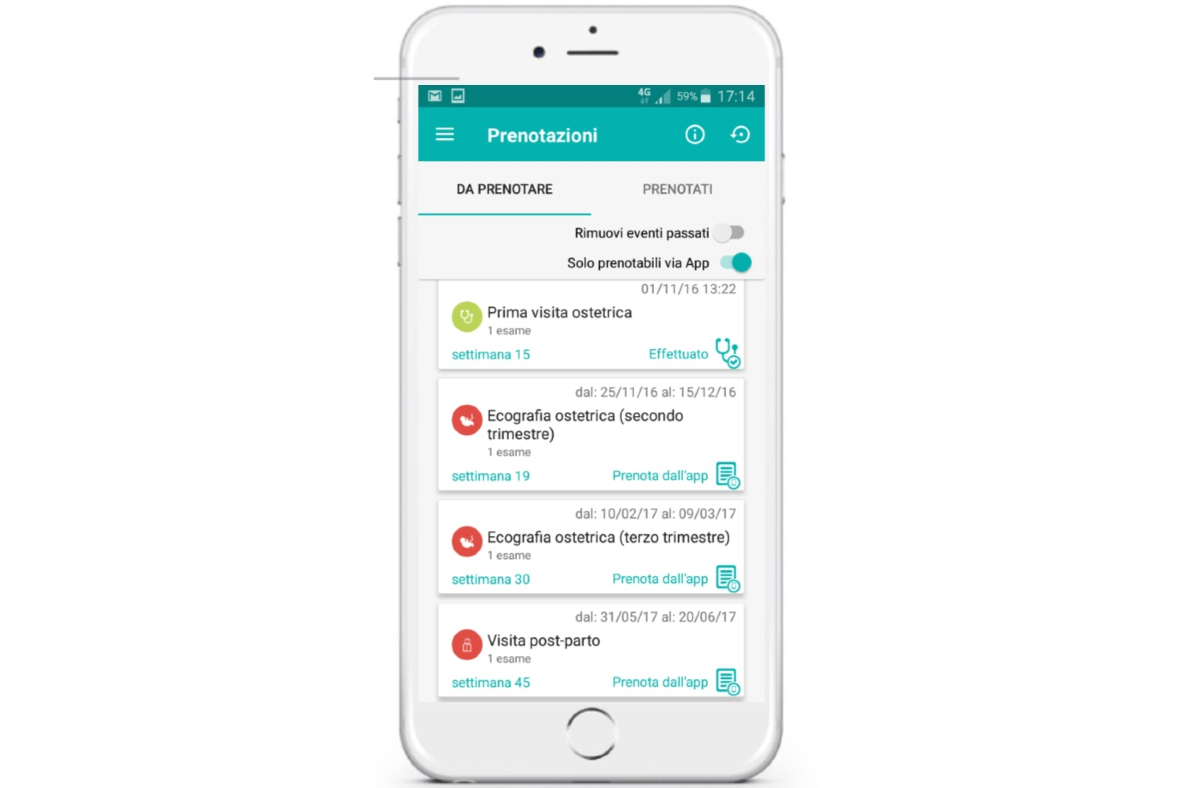
hAPPyMamma e-booking of visits and tests, integration into the app planner, and synchronization with the local health authority booking system.

Women can digitally book, directly from the app, the 3 obstetric ultrasounds (the first visit in pregnancy and the postpartum visit; Figure 3), which are the mandatory touchpoints of the maternal care pathway in Tuscany.

**Figure 4:**
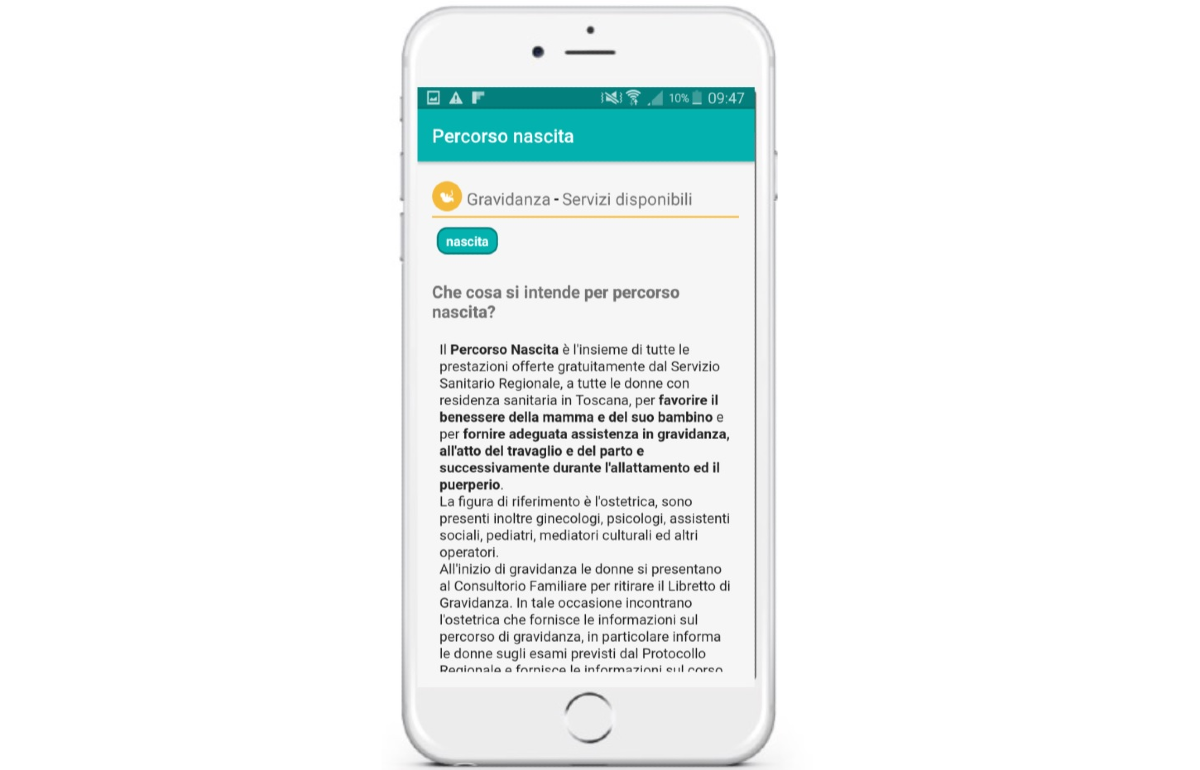
hAPPyMamma information repository: provision of professionally validated information (in a frequently asked questions format) on health promotion, prevention, and health care services concerning pregnancy, childbirth, and the postpartum period, with information proactively suggested via pop-up messages.

hAPPyMamma contains information on health promotion and prevention as well as on the health care services concerning pregnancy, childbirth, and the postpartum period, divided into thematic sections (Figure 4). It also proactively shows information through pop-up messages based on data reported by women (ie, smokers will receive pop-up messages on the specific topic of smoking during pregnancy).

**Figure 5:**
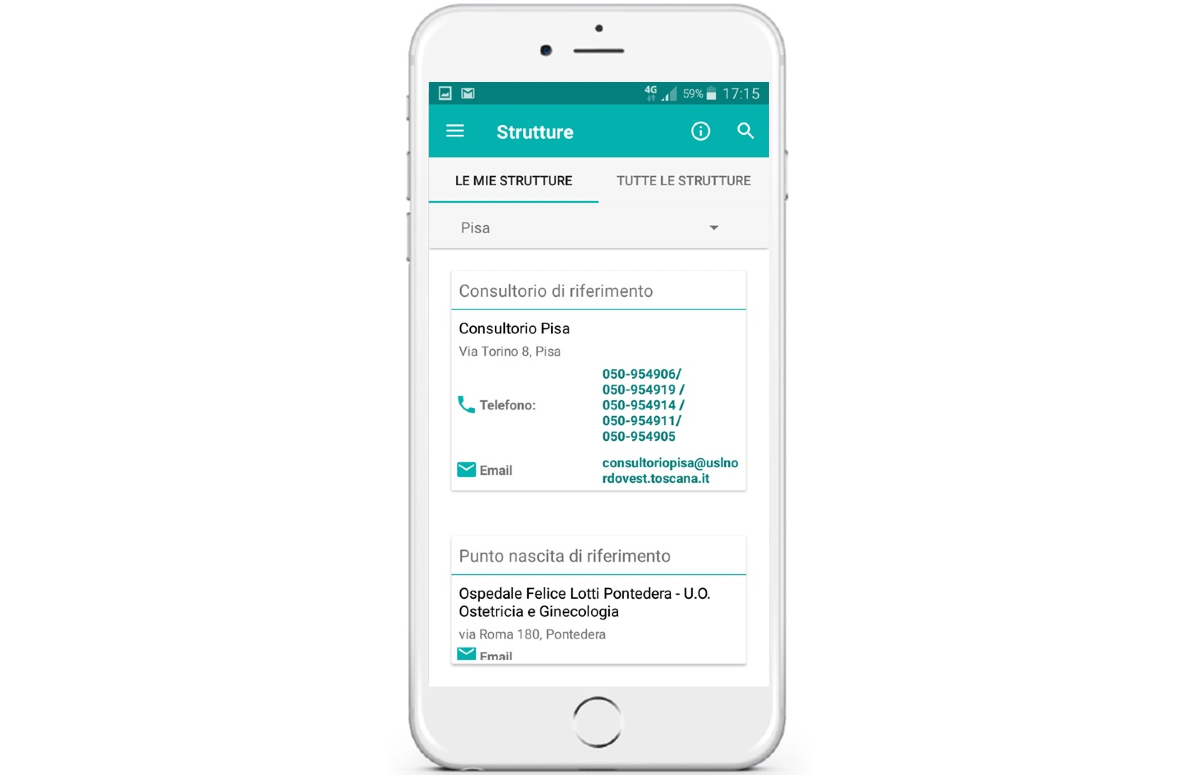
hAPPYMamma facilities repository: information on family care centers and delivery hospitals, with logistic details, services provided, and georeferencing system embedded.

hAPPyMamma includes a section with logistic details of primary care and hospital services along the maternity care pathway, with a georeferencing system (Figure 5). It first presents services and providers related to the woman’s residence area. Additionally, hAPPyMamma is integrated with the regional mobile app that allows access to personal health records developed by the Tuscan Regional Health System. Finally, women using the app can provide direct feedback and answer questionnaires proposed by the app to evaluate their experience in the maternity care pathway.

The design of hAPPyMamma was user-driven, as described, and its development was shared with the professionals involved at both the primary care and hospital levels and in both the maternal and child care areas. It involved the researchers of the Sant’Anna School, who facilitated the app design process and evaluated the results of this innovation.

### Objectives

The aim of the study reported in this protocol is to evaluate whether and to what extent the mobile app hAPPyMamma is able to increase maternal health literacy (MHL) and empowerment of women as well as access to and utilization of health care services during the maternal care pathway. Our hypothesis is that implementation of the mHealth solution will bring a positive impact on the maternal care pathway, in terms of more appropriate use of the available services, a better experience for women, and an improvement in the maternal competencies of women using hAPPyMamma. To meet these objectives, the study proposes to compare prestudy and poststudy differences in outcomes for the control group versus the intervention group using hAPPyMamma.

Moreover, the protocol includes an analysis of the use of hAPPyMamma for the intervention group and an analysis of the organizational impact of the introduction of the app in the maternal pathway from the professionals’ perspectives.

## Methods

Impact evaluation of the hAPPyMamma mHealth intervention is provided in detail in this section.

### Study Design

In order to assess the impact of the use of hAPPyMamma in the maternal care pathway, this study uses a pre-post quasiexperimental design that compares 2 groups: women who use the app (intervention group) and women who do not use the app (control group). The manipulation of the independent variable (app use) is done by recruiting the 2 groups consequentially: control group before the app was available on the app stores and the experimental group when the app was introduced into the maternal care pathway. The ethical committee of the LHA where the mHealth intervention is implemented approved the research protocol (Authorization n. 42379 signed by the ethical committee on July 13, 2016 and registered with the approval number n. 0133972 on August 3, 2016).

The choice of not randomizing the samples using and not using the mobile app is in line with the ethical principles. Indeed, the mobile app can represent a potential tool to improve the quality of the maternity care pathway, and the randomization of women could have brought unfair advantages to women who use the app compared to the others. Therefore, the choice of a quasiexperimental study design also addresses this ethical principle.

The data concerning the measures of maternal health literacy and empowerment are collected prospectively through web-based prestudy and poststudy surveys, enabling difference-in-differences analysis to assess the impact of the mHealth intervention [42]. In particular, there are 2 data collection moments through the web platform. The first data collection (prestudy survey) is carried out at the beginning of the maternal care pathway, when women receive the pregnancy booklet, corresponding to the beginning of the pregnancy. The second data collection (postsurvey) is implemented around 6 months after childbirth in the postpartum period, when data on women’s experiences in the maternal care pathway are also collected. This second survey questionnaire also contains a section reserved for the intervention group on their experience with hAPPyMamma use.

The organizational impact will be evaluated through a quantitative and qualitative survey and in-depth interviews addressing professionals and managers of the maternal care pathway in the participating LHA and teaching hospital. Both data collection methods focus on the perceived changes the practitioners identify in their job activities due to the mHealth intervention. The survey uses a web questionnaire with close-ended and open-ended questions, in order to measure and assess practitioners’ perceptions and opinions on the hAPPyMamma impact through rating questions and narratives. The survey results are discussed with some practitioners during the in-depth interviews. Considering that the organizational impact can be better evaluated through a midterm evaluation, this component of the study protocol is planned to be implemented after implementation of the data collection with mothers.

### Study Population and Sample Size

The sample size for the pre-post survey addressing mothers was estimated with respect to differences concerning some key measures considered within the study, namely MHL level and other experience measures such as access to maternal care services and satisfaction with the maternal care received. We determined the sample size required to detect a 10% difference between the 2 groups concerning the key measures considered within the study, when this difference genuinely exists in the populations of the mothers, with a power of 80% and an alpha error of 5%. With these parameters, a sample size of 300 pregnant women, divided equally between the control and intervention groups, was considered appropriate for a study population of around 2000 births in the pilot LHA yearly.

The quantitative and qualitative survey focusing on the organizational impact of the mHealth intervention uses a convenience sample of professionals and managers working in the LHA and teaching hospital of the study area. All midwives, obstetricians, pediatric doctors, and other professionals of the maternal care pathway are invited to fill in the web questionnaire. The results of this data collection are discussed with 15 practitioners during in-depth interviews.

### Eligibility Criteria

All women receiving the pregnancy booklet in the family care centers of the 3 districts of the participating pilot LHAs during the recruitment period were included. The only exclusion criterion is not speaking Italian, because the app does not have the multilanguage function activated during the experimentation phase. Finally, women without smartphones are not able to use hAPPyMamma and thus are unable participate in the intervention group of the study.

### Outcomes

The study objectives are addressed in the following ways.

#### Difference in Improvements in MHL and Empowerment Between the Control and Intervention Groups

Key outcomes of the study concern MHL and empowerment of women involved in the maternity pathway, which may be particularly improved in the intervention group thanks to the use of the mobile app hAPPyMamma. In order to measure these outcomes, some items of internationally validated tools are used [43-45]. Table 1 shows the dimensions included in the MHL construct: critical, functional, self-efficacy, and social capital. The first 3 dimensions of MHL focus mainly on underlining the competence of mothers for promoting and protecting their health and that of their children, as well as orienting among health information and services. The fourth dimension concerns social capital that can be considered both a demonstration and a consequence of MHL. In order to define the MHL items for the pre-post questionnaire, 2 different researchers translated the items from the international scales, compared and discussed the 2 different translations, and finally identified a shared version of the Italian items.

Empowerment is evaluated in our study in terms of self-efficacy on breastfeeding and duration of breastfeeding (total and exclusive). These measures that are internationally considered a proxy indicator of mother’s empowerment can be positively affected by the more direct and easy access to information on breastfeeding through the app.

**Table 1:** Dimensions included in the pre-post survey questionnaires addressing sampled women in the maternal care pathway.

Dimensions		Description	Control group		Intervention group	
			Prestudy survey	Poststudy survey	Prestudy survey	Poststudy survey
Maternity pathway expectations		Expectations about pregnancy, delivery, and the postpartum period (1 item)	X	N/A^a^	X	N/A
**Maternal health literacy**						
	Critical	Search of different sources of information, check of validity and reliability of information, use of information to make decisions on own health (7 items)	X	X	X	X
	Functional	Ability to understand health information, difficulty in reading and interpreting health information materials, self-confidence to fill in modules with health information (4 items)	X	X	X	X
	Self-efficacy	Self-confidence to follow health indications, self-confidence to be autonomous in taking care of own child, capability to identify different positive solutions facing obstacles (4 items)	X	X	X	X
	Social capital	Support from someone in case of concerns or doubts on own condition, continuity of social life after pregnancy (2 items)	X	X	X	X
Intention to breastfeed		Expectations of breastfeeding and its duration (2 items)	X	N/A	X	N/A
Breastfeeding - empowerment		Total breastfeeding, exclusive breastfeeding, and its duration (3 items)	N/A	X	N/A	X
Experience in the maternal care pathway		Booking of exams during pregnancy, awareness of prenatal diagnostic tests, sources of information on prenatal diagnosis tests, attendance at antenatal classes, difficulties in accessing health services during pregnancy, awareness of labor and delivery, use of health services after delivery, orientation difficulties in maternal care pathway, satisfaction with the maternal care received, suggestions on maternal care pathway improvement (14 items)	N/A	X	N/A	X
Type of health care during pregnancy and delivery		Professionals and services involved in the pregnancy follow-up, number of visits and ultrasounds, characteristics of pregnancy and delivery (8 items)	N/A	X	N/A	X
Interest in ICT^b^ services		Information and reminders for visits and exams, antenatal classes, and postpartum services; information on healthy lifestyle during pregnancy; health information records for pregnancy, delivery, and the postpartum period; communication with family pediatrician—by different ICT tools (8 items)	N/A	X	N/A	X
Experience with hAPPyMamma use		Duration and frequency of use, habits in smartphone and internet use, comparison with other apps concerning maternal care, utility of different app functionalities, suggestions to improve the app use (11 items)	N/A	N/A	N/A	X
Usability of hAPPyMamma		Learnability, memorability, understandability, attractiveness, errors, efficiency, evaluation of quality, satisfaction in terms of willingness to recommend (15 items)	N/A	N/A	N/A	X
Sociodemographic characteristics		Age, citizenship, education level, employment status, gestational age, number of children (6 items)	X	N/A	X	N/A

^a^N/A: not applicable.

^b^ICT: information and communication technology.

#### Control and Intervention Group Experiences in the Maternal Care Pathway

The experience of women in the maternal care pathway is measured through a selection of the questions included in the validated questionnaires that have been used periodically in the Tuscany Region to evaluate the users’ perspective in the maternal care pathway [35,36]. As shown in the Table 1, this dimension of experience concerns the use of health services during the maternal care pathway, such as prenatal care, antenatal classes, and postpartum care, and they explore particularly the difficulties in access to and orientation among the health services of the maternal care pathway. The questions focus also on the perceived quality of the maternal care pathway and the willingness to recommend its services to friends and family members. The use of the same survey tool ensures continuity in the research approach and the possibility to assess the trend in the outcomes measured.

#### Intervention Group’s Use of hAPPyMamma

A specific section concerning the experience of the hAPPyMamma use and its usability is introduced in the post-survey questionnaire for the intervention group only (Table 1). The first dimension explores mainly the duration and frequency of the use of hAPPyMamma, perceived utility of the hAPPyMamma functionalities, and opinions of mothers concerning the comparison of hAPPyMamma with other apps related to maternity. The usability includes the principal criteria included in the scales used at the international level [46-49], such as learnability, memorability, understandability, attractiveness, errors, efficiency, quality, and satisfaction. Moreover, some questions focusing on the mothers’ interest for ICT services are introduced in the post-survey questionnaire; these investigate mothers’ possible interest in receiving—by email, message, or app—information on and reminders for visits and exams, antenatal classes, and postpartum services; information on a healthy lifestyle during pregnancy; health information records for pregnancy, delivery, and the postpartum period; and communication with the family pediatrician. They are submitted both to intervention and control groups in order to also assess the interest of mothers who are not using the mHealth solution. The intervention group has an additional option for answering these questions, namely that they already receive information and reminders via hAPPyMamma.

#### Organizational Impact of the Introduction of the hAPPyMamma App in the Maternity Pathway From the Professionals’ Perspectives

The organizational impact is assessed through a web survey and in-depth interviews with the professionals and managers of the maternal care pathway (midwives, obstetricians, neonatal doctors), focusing on the aspects of the experimental use of the mobile app affecting the health care process. In particular, the web survey and interviews explore their expectations before mHealth solution implementation, their perceived difficulties and worries, feelings of uncertainty, fear of replacement, desire to innovate, and the resistance to change that may characterize the opinions of the involved professionals. The descriptive analysis of the questionnaire allows elucidation of the experience of professionals and managers regarding the mHealth intervention. These results represent the basis of the discussion with some professionals and managers during the in-depth interviews. The implementation of the web survey and in-depth interviews after the end of the experimentation allow description of the impact of hAPPyMamma on health care services and practices from the points of view of professionals, with a certain period of distancing from the mHealth intervention to better appreciate its organizational impact.

### Statistical and Qualitative Data Analysis

Descriptive statistics will be used to characterize the study population to give an overview of the 2 groups concerning demographic characteristics and use of services during pregnancy, birth, and the postpartum period.

The impact of the app hAPPyMamma on the MHL will be evaluated through a difference-in-differences analysis, which allows measuring the difference in changes prestudy and poststudy between the 2 groups. In particular, the panel data of the study are used to measure the differences between the treatment and control group in the changes in the outcome variable (MHL) that occur over time. In particular, we will calculate the effect of the use of hAPPyMamma (ie, an explanatory variable or an independent variable) on MHL (ie, a response variable or dependent variable) by comparing the average change over time in the outcome variable for the treatment group and the average change over time for the control group. We will also verify the impact of the use of hAPPyMamma on the subdimensions of MHL.

Other multivariate analyses of variance will be carried out in order to evaluate empowerment related to breastfeeding results and the experience of women in the maternity pathway, specifically comparing the control and intervention groups.

Descriptive statistics and multivariate models will also be used to analyze the experience with hAPPyMamma use and its usability.

All statistical analyses are performed using SAS and Stata software.

Descriptive statistics will be performed for the web questionnaires completed by professionals and managers. These results will be presented to key practitioners involved in the in-depth interviews. Qualitative analysis of the in-depth interviews will be performed using QSR NVivo software. After importing the narrative answers to the web questionnaire and the transcriptions of the in-depth interviews, data coding will be implemented by 2 researchers. Content analysis will be carried out in order to identify emerging themes and patterns of the perceptions of professionals on the organizational impact of hAPPyMamma in the maternal care pathway [50].

## Results

Recruitment of the 2 samples of mothers (control and intervention groups) was carried out in sequence. Data collection with the control group started at the beginning of 2017. In May 2017, hAPPyMamma was made available on app stores, and recruitment of the intervention group started. Both groups were recruited using the same procedures. At the time of receiving the pregnancy booklet, women were informed about the study by midwives. Women who decided to participate signed a consent form, leaving their email address. For the control group, we sent an invitation by email to fill in the first web questionnaire at the beginning of the maternity pathway, while the intervention group received the invitation concerning the first web questionnaire directly from the app. The invitation for the second questionnaire was sent by email to both groups, with the intervention group also having the possibility to access the web questionnaire from the app.

The participation rate was high for both the control and intervention groups (around 97%-98%), since the consensus for attending the study was given by 177 women in the former group and by 150 women in the latter group. The response rate for the first questionnaire was different between the 2 groups: 96% (170/177) in the control group and 67% (100/150) in the intervention group. The difference in respondent loss in the follow-up questionnaires was reversed: 33% (56/170) in the control group and only 10% (10/100) in the intervention group. Data collection was completed in April 2018.

Data analysis as well as data collection with professionals and managers are currently underway.

## Discussion

This paper provides the protocol to evaluate the implementation of an mHealth intervention and its impact at individual and organizational levels in terms of improvement in maternal health literacy, mothers’ empowerment, and access to and utilization of health care services in the maternal care pathway. We are collecting data to describe the experimental use of the app hAPPyMamma and report on benefits for the mothers using the app. This study is innovative in the Italian context and compared with other interventions worldwide. The mHealth intervention has been realized thanks to collaboration between university researchers and health professionals in maternal care and is promoted by the Regional Health Authority with the aim of improving the quality of the maternal care pathway. Therefore, the study results assume an institutional perspective and will provide insights on the impact of hAPPyMamma use from the organizational as well as the user perspectives and on the perceptions on the provision of several services in the maternal care pathway through this mHealth channel.

Interviews with key professionals in the maternal care pathway will help to deeply understand their point of view alongside insights emerging from the quantitative and qualitative web survey. This will contribute to identifying and explaining factors positively and negatively affecting the implementation and deployment of hAPPyMamma from their perspectives and those that may facilitate or inhibit the normalization of the innovative tool within the maternal care pathway.

The findings of this study will be relevant for the academic community as well as for policy makers and practitioners. First, there is scarce empirical evidence of the real potential of mHealth in improving women’s access to care, their literacy and self-management skills, and quality of services along the maternal pathway [9]. This is particularly true for western countries because the literature focuses on the impact of technologies in developing countries [14-29]. In high-income countries, technology-supported interventions targeted at pregnant women and new mothers are often aimed at improving their lifestyle-related behaviors [9,51]. Conversely, the current critical circumstances that impose social distancing and limit physical access to care have highlighted the need for evidence-based technologies to be introduced to support digital and at-distance health care services in a time of crisis, which could be maintained in normal times. This is the second key point that supports the need for evidence on the effectiveness of mHealth services for women in the maternal pathway in developed countries. Service innovation is urgently needed in health care [52], and hAPPyMamma is an innovative way of providing women-centered services along a pathway, also allowing the evaluation of several different outcomes.

There are several strengths in the design of this study. Its methodological approach as a quasiexperimental study allows overcoming the limitations of observational studies in measuring the effectiveness of interventions and their impact at the individual and organizational levels [53,54]. It is an appropriate method for evaluating policies or interventions, such as hAPPyMamma, collecting data before the recipients are exposed to policy or intervention activities [55]. The results of the study contribute to verifying the possibilities and potential of the scaling up of the mHealth intervention.

The study faces some potential methodological and practical challenges. The nonrandomization of the sample, which is an important aspect from an ethical point of view, represents a weakness of the study. Indeed, the noncontemporaneous recruitment of the intervention and control groups does not allow excluding the possibility of influencing factors due to environmental or organizational context [54,56].

Moreover, the differences in response rate and loss to follow-up among maternal samples have to be taken into account, and data analysis will verify if these affect the results [57].

In conclusion, this study contributes to defining the potential role of the mHealth intervention hAPPyMamma in the maternal care pathway. The findings of this study could provide valuable insights on the benefits of hAPPyMamma use for women’s experiences in maternal care pathways. Therefore, this study could significantly support analysis to understand if scaling up hAPPyMamma implementation from the pilot area to the Tuscany Region, as well as to the entire country, would be beneficial. As anticipated, the findings that will result from the evaluation of this mHealth intervention will also provide useful insights for supporting the introduction of mobile-based innovations in maternal and newborn care pathways in other (developed as well as developing) countries.

## Abbreviations

ICT: information and communication technology
LHA: local health authority
MHL: maternal health literacy
